# Systematic review and meta-analysis on trimodal therapy versus radical cystectomy for muscle-invasive bladder cancer: Does the current quality of evidence justify definitive conclusions?

**DOI:** 10.1371/journal.pone.0216255

**Published:** 2019-04-29

**Authors:** Marian S. Wettstein, Jasjit K. Rooprai, Clinsy Pazhepurackel, Christopher J. D. Wallis, Zachary Klaassen, Elizabeth M. Uleryk, Thomas Hermanns, Neil E. Fleshner, Alexandre R. Zlotta, Girish S. Kulkarni

**Affiliations:** 1 Division of Urology, Department of Surgery, Princess Margaret Cancer Centre, University Health Network, University of Toronto, Toronto, Ontario, Canada; 2 Department of Urology, University Hospital of Zurich, University of Zurich, Zurich, Switzerland; 3 E.M. Uleryk Consulting, Mississauga, Ontario, Canada; 4 Division of Urology, Department of Surgery, Mount Sinai Hospital, Sinai Health System, University of Toronto, Toronto, Ontario, Canada; University of Mississippi Medical Center, UNITED STATES

## Abstract

**Objectives:**

To systematically review and meta-analyze the current literature in a methodologically rigorous and transparent manner for quantitative evidence on survival outcomes among patients diagnosed with muscle-invasive bladder cancer that were treated by either trimodal therapy or radical cystectomy.

**Materials and methods:**

MEDLINE, EMBASE, CENTRAL were systematically searched for comparative observational studies reporting disease-specific survival and/or overall survival on adult patients diagnosed with localized muscle-invasive bladder cancer that were exposed to either trimodal therapy or radical cystectomy. Studies qualified for meta-analysis (random effects model) if they were not at critical risk of bias (RoB).

**Results:**

The literature search identified 12 eligible studies. Three (all rated as “moderate RoB”) out of 6 studies reporting on disease-specific survival qualified for quantitative analysis and yielded a pooled hazard ratio (trimodal therapy versus radical cystectomy) of 1.39 (95% confidence interval: 1.03–1.88). Four (mainly rated as “serious RoB”) out of 12 studies were included in the meta-analysis of overall survival and estimated a hazard ratio of 1.39 (1.20–1.59).

**Conclusion:**

Pooled results were significant in favor of radical cystectomy. The conclusion is mainly driven by large population-based studies that are at high RoB. Hence, the certainty of these treatment estimates can be considered very low and further research will likely have an important impact on these estimates. At present, the ultimate decision between trimodal therapy and radical cystectomy should be left to the patient based on individual preferences and on the recommendation of a multidisciplinary provider team experienced with both approaches.

## Introduction

Radical cystectomy (RC) is considered the gold-standard therapy for localized muscle-invasive bladder cancer (MIBC) as demonstrated by large series with long-term follow-up [1, 2]. However, this procedure is associated with both a substantial decrease in the postoperative quality of life [3, 4] and also high rates of postoperative morbidity and mortality, estimated to be as high as 64% and 2.7%, respectively, even in tertiary referral centers [5]. Especially in the subpopulation of high-risk surgical patients and in appropriately selected individuals seeking to preserve their native bladder, bladder-sparing treatment options have been investigated as an alternative curative treatment to RC. A trimodal approach consisting of a maximal transurethral resection of the bladder tumor (TURBT) followed by radiation therapy (RT) and concurrent chemotherapy is currently considered to yield the best oncologic results among bladder-sparing treatment modalities [6].

While a randomized controlled trial demonstrated superiority of trimodal therapy (TMT) over bimodal treatment (TURBT followed by RT) for patients with MIBC [7], randomized controlled evidence comparing TMT and RC is currently neither available nor anticipated [8]. When choosing one of these 2 options, treating physicians and their patients are left with a heterogeneous body of evidence consisting of few comparative observational studies. Recently published systematic reviews and meta-analyses on this research question lack methodological rigor and transparency with regard to study selection, comprehensive risk of bias assessment and appropriate quantitative synthesis [9–11]. Further, they yielded discrepant results. In the absence of a contemporary and internally valid evidence synthesis, we aimed to systematically review the literature in a methodologically rigorous and transparent manner for quantitative comparative evidence regarding survival outcomes, namely disease-specific survival and overall survival, among adult patients diagnosed with MIBC who were treated either by TMT or RC.

## Materials and methods

### Registration, reporting and eligibility criteria

The methods of this systematic review and meta-analysis were specified in advance in a protocol and the reporting follows the PRISMA 2009 Checklist (Preferred Reporting Items for Systematic Reviews and Meta-Analysis) [12]. The protocol was prepared in concordance with the PRISMA-Protocol 2015 Checklist [13] and registered with PROSPERO (International Prospective Register of Systematic Reviews) on February 19, 2018 (registration number: CRD42018086589; last update: March 1, 2018). *Studies*: In the absence of randomized controlled trials (RCT) comparing TMT and RC for MIBC regarding survival outcomes, non-randomized comparative studies (NRS) were considered as source of evidence as defined in *S1 Text*.

#### Population

Studies involving adult patients (>18 years) diagnosed with localized urothelial MIBC were considered regardless of the multiplicity and/or size of the tumor. We excluded studies involving *exclusively* non-adult patients (<18 years), locally advanced disease (cT4b and/or cN1+), systemic disease (M1), non-urothelial histology (predominant), prior trimodal therapy (as defined later) and/or prior pelvic irradiation regardless of the disease site. *Intervention*: We defined TMT as maximal TURBT followed by RT and concurrent chemotherapy regardless of RT dose, type/dose of concurrent chemotherapy, salvage RC regimen, continuous or split course (see *S1 Fig*), neoadjuvant chemotherapy (NAC) or adjuvant chemotherapy (AC). We excluded studies describing: local resection methods other than monopolar/bipolar TURBT, non-external beam RT, intraarterial chemotherapy, tyrosine-kinase inhibitors, check-point inhibitors or heterogeneous bladder-sparing arms without reporting of isolated TMT outcomes.

#### Comparison

Studies having RC as a comparison arm were considered eligible regardless of the surgical approach (open/laparoscopic/robot-assisted), type of urinary diversion, extent of pelvic lymph node dissection (PLND), NAC/AC or neoadjuvant/adjuvant radiation therapy. Studies exclusively involving simple cystectomy or RC without PLND were not considered eligible.

#### Outcomes

Outcomes of interest were disease-specific survival [DSS; defined as time to death due to bladder cancer] and overall survival [OS; defined as time to death due to any cause]. Studies were included if they presented a hazard ratio (HR) and/or a Kaplan-Meier curve.

#### Timing, setting, language

Neither restrictions to follow-up time, setting nor language were applied.

### Information sources, search, study selection, data collection and data items

MEDLINE (OvidSP), EMBASE (OvidSP) and CENTRAL (Wiley) were searched on February 10, 2018 by a sensitive search strategy (see *S1 Table*) developed by a health science librarian with extensive expertise in systematic reviews (EMU). The last search update took place on August 1, 2018. More information on the development of the search strategy, study selection, data collection as well as on data items can be found in *S1 Text*.

### Risk of bias assessment

The risk of bias (RoB) was evaluated by the ROBINS-I tool (Risk Of Bias In Non-randomized Studies–of Interventions) at the outcome level by two independent reviewers (MSW, CJW or ZK) [14]. Disagreements were resolved by discussion with a third reviewer (MSW, CJW, ZK or GSK) and audited by the complete team. The evaluation was guided by an iteratively developed framework that not only incorporates the requirements for a corresponding target trial but also outlines the most relevant confounding themes based on a causal diagram and defines warranted adjustment factors (see *S2 Fig* and *S1 Text* for more details).

### Synthesis of results

The accumulated evidence was first qualitatively synthesized to allow an assessment of the heterogeneity of the included studies. The preferred summary measure of the time-to-event outcomes DSS and OS was the HR. Effect estimates and their corresponding standard errors were preferably directly abstracted from the studies or mathematically/graphically derived as described by *Tierney et al*. [15]. Studies were not eligible for meta-analysis if their overall RoB was considered as “critical”. Furthermore, only one population-based study was allowed per database and per time period to ensure that a single patient does not contribute to the summary effect more than once. If investigators used different statistical approaches for analysis, we selected primarily the approach that was least prone to bias and secondarily the most commonly used approach in the remaining body of evidence.

Pooling was performed within the strata “single-center studies only” and “all studies” whereas the latter approach also incorporated population-based studies. The robustness of the conclusions was further verified by sensitivity analyses in which we iteratively exchanged studies whose inclusion/exclusion were at high subjectivity. For meta-analyses a random-effects model according to *DerSirmonian & Laird* [16] was used as we assumed the true effect size of TMT versus RC to be heterogeneous across studies. Pooling was performed according to the generic inverse variance method. Finally, the cumulative evidence was assessed by the GRADE methodology (Grading of Recommendations Assessment, Development and Evaluation) [17]. All quantitative syntheses were performed in R 3.4.4 (The R Foundation, Vienna, Austria) using the *meta* package [18].

## Results

### Study selection

**Fig 1:**
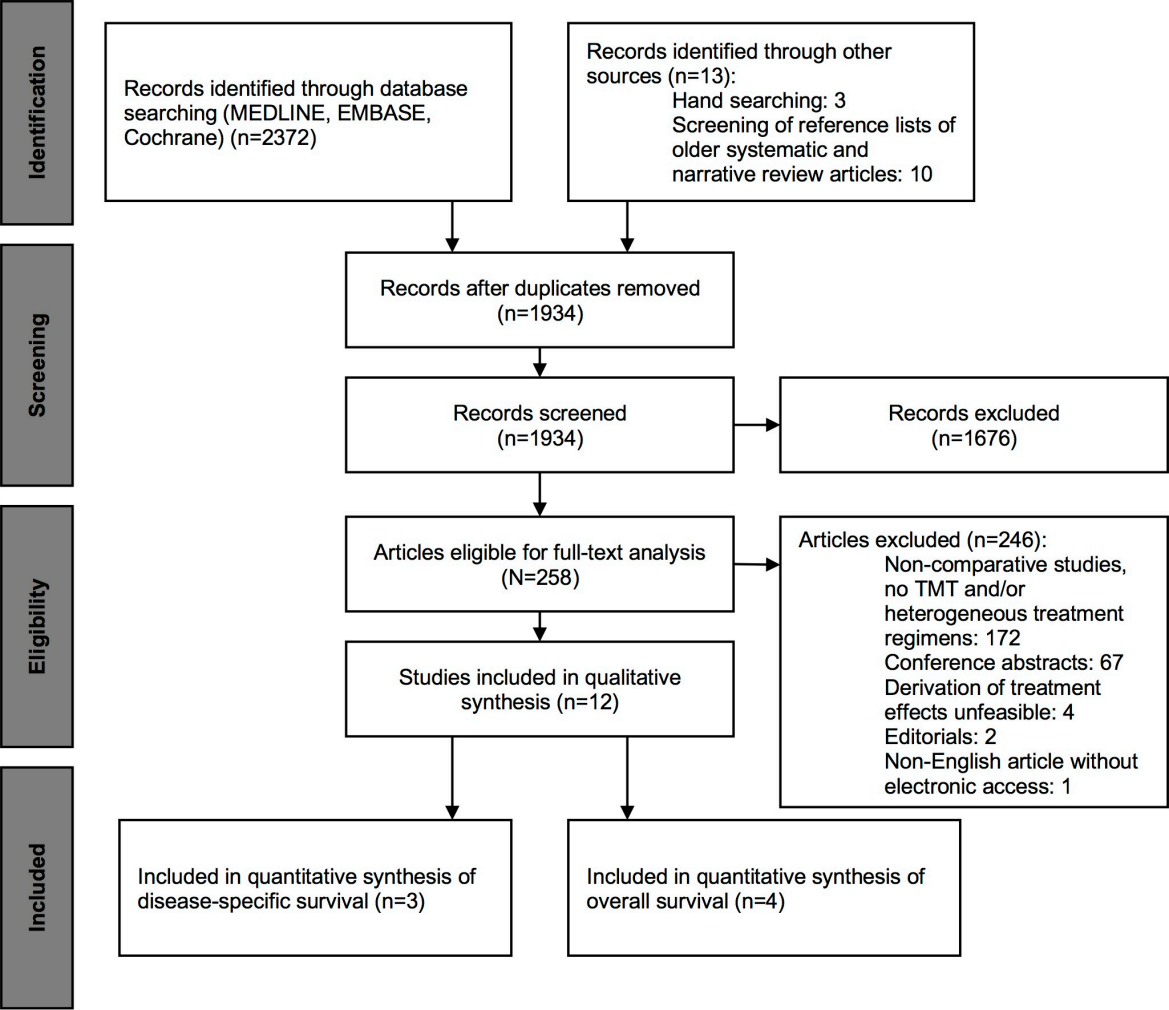
PRISMA (Preferred Reporting Items for Systematic Reviews and Meta-Analysis) flow diagram illustrating the study selection process.

A total of 12 studies were eligible for inclusion into qualitative synthesis. The PRISMA flow diagram of the study selection process is presented in *Fig 1* and described in more details in *S2 Text*.

### Study characteristics

The characteristics of all eligible studies (N = 12) are presented in *Table 1*. All studies were of retrospective nature and recruited patients in the interval between 1995 and 2014 with median follow-up times ranging from 13 months to 54 months. Five eligible studies were single-center studies (Israel: 1 [19], Japan: 2 [20, 21], South Korea: 1 [22], Canada: 1 [23]) while 7 studies were population-based and relied on two partially overlapping United States-based registries (Surveillance, Epidemiology, and End Results [SEER]-Medicare database: 2 [24, 25], National Cancer Database [NCDB]: 5 [26–30]) and hence provided a higher number of patients compared to the single-center studies (N_single-center_: 413, N_SEER-Medicare (max)_: 1843, N_NCDB (max)_: 24169). The majority of the identified studies included cT2-T4/cN0/cM0 patients without any age restrictions. *Ikeda et al*. and *Kulkarni et al*. [20, 23] allowed for cN+ disease. The first study further focussed on more advanced stages (cT3-T4a) while *Bekelman et al*. [24] and *Smith et al*. [29] limited their cohort to only cT2-T3 and cT2 patients, respectively. Three of the population-based studies applied age restrictions. *Fischer-Valuck et al*. [26] limited their cohort to octogenarians while the two SEER-Medicare-based studies [24, 25] allowed only included patients older or equal to 65/66 years (*Bekelman et al*./*Williams et al*.)

**Table 1:** Study characteristics (N = 12).

Study Location	Design Type Center or database N_total_ Accrual interval	Population	Arms (N) including treatment specifications (course, RT dose, concurrent chemotherapy agents, NAC, AC and extent of PLND)	Follow-up (median, months)	Reported outcomes
*Single-center studies*					
Gofrit, 2015 [19] Israel	Retrospective Single center Jerusalem N = 66 1998–2008	cT2-T4a cN0 cM0	TMT arm (33) • Continuous course • 62 Gy • Cisplatin (carboplatin if renal function impaired) • Exclusion: bulky tumor and/or obstructed kidney RC arm (33) • PLND template: common iliac, external iliac, obturator • NAC or AC: no information available	35 (TMT), 36 (RC)	DSS, OS
Ikeda, 2014 [20] Japan	Retrospective Single center Kanagawa N = 72 2002–2012	cT3-T4a cN+ (29) cM0 NUH (4)	TMT arm (40) • Continuous course • 55 Gy • MVAC • NAC: MVAC (1x) • AC: MVAC (2x) RC arm (32) • PLND template: common iliac, external iliac, internal iliac, obturator • AC: no information available • Exclusion: NAC	20 (TMT), 27 (RC)	OS
Kim, 2017 [22] South Korea	Retrospective Single center Seoul N = 340 2007–2014	cT2-T4 cN0 cM0	TMT arm (32) • Continuous course • 60 Gy • Cisplatin • 8 patients did not receive concurrent chemotherapy • NAC (8) or AC (1): gemcitabine-cisplatin x 4 RC arm (308) • PLND template at surgeon’s discretion • NAC (26) or AC (6): gemcitabine-cisplatin x 4	31 (TMT), 43 (RC)	DSS, OS
Kulkarni, 2017 [23] Canada	Retrospective Single center Toronto N = 112 2008–2013	cT2-T4 cN+ (20) cM0	TMT arm (56) • Continuous course • 66 Gy • Cisplatin • NAC (20): gemcitabine-cisplatin x 4 • Exclusion: tumors > 5cm, multiple tumors, more than mild hydronephrosis, multifocal carcinoma in situ, impaired bladder function RC arm (56) • PLND template at surgeon’s discretion • NAC (11) or AC (10): gemcitabine-cisplatin x 4	54	DSS, OS
Nagao, 2017 [21] Japan	Retrospective Single center Ube N = 84 1994–2011	cT2-T4 cN0 cM0 NUH (8)	TMT arm (42) • Continuous course • 49 Gy • Cisplatin RC arm (42) • PLND template: not specified • NAC/AC: not specified	94 (TMT, mean), 54 (RC, mean)	DSS, OS
*Population-based studies*					
Bekelman, 2013 [24] United States	Retrospective Population-based SEER-Medicare N = 1843 1995–2005	cT2-T3 cN0 cM0 ≥65 y	TMT arm (417) • Continuous course or split course with complete response at re-evaluation • RT dose not defined • Platinum-based chemotherapy • NAC/AC: not specified RC arm (1426) • With or without PLND • No NAC/AC	not reported (end of observation period: December 2008)	DSS, OS
Cahn, 2017 [30] United States	Retrospective Population-based NCDB N = 24169 2004–2013	cT2-T4a cN0 cM0	TMT arm (1489) • Continuous course or split course with complete response at re-evaluation • 50–80 Gy • Various single- or multiple agent chemotherapy • NAC/AC: not specified RC arm (22680) • With or without PLND • NAC/AC: not specified	not reported (end of recruitment period: December 2013)	OS
Fischer-Valuck, 2018 [26] United States	Retrospective Population-based NCDB N = 2279 2004–2013	cT2-T4a cN0 cM0 80–90 y	TMT arm (958) • Continuous course or split course with complete response at re-evaluation • ≥50 Gy • Various single- or multiple agent chemotherapy • NAC/AC: not specified RC arm (1231) • With or without PLND • No NAC/AC	13	OS
Ritch et al., 2018 [27] United States	Retrospective Population-based NCDB N = 3366 2004–2013	cT2-T4 cN0 cM0 NUH (1244)	TMT arm (1686) • Continuous or split course • ≥40 Gy • Various single- or multiple agent chemotherapy • NAC or AC: not specified RC arm (1686) • With or without PLND • NAC/AC: not specified	45	OS
Seisen et al., 2017 [28] United States	Retrospective Population-based NCDB N = 12843 2004–2011	cT2-T4 cN0 cM0	TMT arm (1257) • Continuous or split course • 60–65 Gy (or ≥39 Gy if followed by immediate salvage RC) • Various single- or multiple agent chemotherapy • NAC/AC: not specified RC arm (11586) • With or without PLND • NAC/AC: not specified	44	OS
Smith et al., 2014 [29] United States	Retrospective Population-based NCDB N = 13428 1998–2010	cT2 cN0 cM0	TMT arm (3724) • Not specified RC arm (9704) • With or without PLND • NAC/AC: not specified	33 (TMT), 38 (RC)	OS
Williams et al., 2018 [25] United States	Retrospective Population-based SEER-Medicare N = 1374 2002–2011	cT2-T4a cN0 cM0 ≥66 y	TMT arm (687) • Continuous course or split course with complete response at re-evaluation • 60 to 66 Gy • Chemotherapy containing cisplatin or fluorouracil + mitomycin C • NAC/AC: not specified RC arm (687) • With or without PLND • NAC (99) • AC: not specified	not reported (claims data available until December 2013)	DSS, OS

AC, adjuvant chemotherapy; DSS, disease-specific survival; Gy, Gray; IPTW, inverse probability treatment weighting; N, number of patients; NAC, neoadjuvant chemotherapy; NCDB, National Cancer Database; NUH, non-urothelial histology; OS, overall survival; PLND, pelvic lymph node dissection; RC, radical cystectomy; RT, radiation therapy; SEER, Surveillance, Epidemiology, and End Results; TMT, trimodal therapy.

All single-center studies described *continuous-course* TMT regimens including platinum-based chemotherapy, radiation therapy doses ranging from 49 to 66 Gray (Gy) and various use of NAC/AC. Two studies [19, 23] further mentioned specific criteria rendering patients TMT-ineligible. Of the population-based studies, only *Ritch et al*. and *Seisen et al*. allowed for capturing of both *continuous-course* and *split-course* TMT regimens by way of definition of the required radiation therapy doses. The remaining population-based studies were restricted by design to *continuous-course* regimens and *split-course* regimens with complete response at re-evaluation. All population-based studies allowed for various chemotherapy regimens and information on the use of NAC/AC was not provided. With regards to the RC arms, data on the extent/utilization of PLND and on the delivery of NAC/AC was sparse although the single-center studies provide more information than the population-based ones. The analytic strategies used in the individual studies and the corresponding effect estimates are presented in *Table 2*. The effect estimates and standard errors of the 3 studies that used Kaplan-Meier analysis were derived graphically [19, 21, 26].

**Table 2:** Analytic strategies used in individual studies and the corresponding effect estimates (hazard ratio_TMT versus RC_).

Study Location	N_TMT_	N_RC_	Analysis	DSS [HR (95% CI)]	OS [HR (95% CI)]
*Single-center studies*					
Gofrit, 2015 Israel	33	33	Hard matching followed by Kaplan-Meier analysis	0.81 (0.31–2.12)*	0.95 (0.38–2.37)*
Ikeda, 2014 Japan	40	32	1. Multivariable regression analysis		1.63 (0.72–3.69)
			2. Propensity score-adjusted regression analysis		1.55 (0.69–3.49)
Kim, 2017 South Korea	32	308	1. Multivariable regression analysis		0.87 (0.39–2.03)
	29	50	2. Propensity score matching followed by adjusted regression analysis	0.96 (0.38–2.47)	0.89 (0.47–2.03)
Kulkarni, 2017 Canada	56	56	Propensity score matching followed by adjusted regression analysis (DSS: accounting for competing risks)	0.92 (0.41–2.04)	0.85 (0.43–1.66)
Nagao, 2017 Japan	42	42	Propensity score matching followed by Kaplan-Meier analysis	0.61 (0.27–1.36)*	0.54 (0.26–1.11)*
*Population-based studies*					
Bekelman, 2013 United States	417	1426	1. Multivariable regression analysis	1.28 (0.98–1.68)	1.26 (1.07–1.50)
			2. Propensity score-adjusted regression analysis	1.31 (0.97–1.77)	1.26 (1.05–1.53)
			3. Inverse probability weighting-adjusted regression analysis	1.34 (1.02–1.77)	1.27 (1.06–1.53)
			4. Instrumental variable analysis	0.94 (0.55–1.18)	1.06 (0.78–1.31)
Cahn, 2017 United States	1489	22680	1. Multivariable regression analysis		1.58 (1.47–1.69)
	1489^1^	1489^1^	2. Hard and propensity score matching followed by unadjusted regression analysis		1.40 (1.24–1.60)
Fischer-Valuck, 2018 United States	958	1231	1. Multivariable regression analysis		0.92 (0.83–1.01)
	650	650	2. Propensity score matching followed by Kaplan-Meier analysis		0.99 (0.88–1.13)*
Ritch, 2018 United States	1686	1686	Propensity score matching followed by adjusted regression analysis		1.5 (1.2–1.8)
Seisen, 2017 United States	1257	11586	Inverse probability weighting-adjusted regression analysis		1.37 (1.16–1.59)
Smith, 2014 United States	3724	9704	Multivariable regression analysis		1.05 (0.98–1.12)
Williams, 2018 United States	687	687	Propensity score matching followed by unadjusted regression analysis (DSS: accounting for competing risks)	1.55 (1.32–1.83)	1.49 (1.31–1.69)

CI, confidence interval; DSS, disease-specific survival; HR, hazard ratio; N, number of patients; OS, overall survival; RC, radical cystectomy; TMT, trimodal therapy.

*Graphical derivation.

^1^assumed.

### Risk of bias assessment

The results of the RoB assessment (overall and stratified by RoB domains) are presented in *Table 3* at the outcome level. One out of 6 studies reporting on DSS was at critical RoB while 2 out of 12 studies reporting on OS were at critical RoB. Details of the comprehensive RoB assessment are presented in *S2 Text*, *S3 Fig* (domain: confounding) and *S4 Fig* (domain: selection bias).

**Table 3:** Risk of bias assessment at the outcome level.

Study	Outcome	Domains							Overall
		Confounding	Selection of participants	Classification of intervention	Deviations from intended intervention	Missing data	Measurement of outcomes	Selection of the reported results	
*Single-center studies*									
Gofrit, 2015	DSS	**critical**	low	low	low	low	low	*moderate*	**critical**
	OS	**critical**	low	low	low	low	low	*moderate*	**critical**
Ikeda, 2014	OS	serious	*Ø* information	low	low	low	low	*moderate*	serious
Kim, 2017	DSS	*moderate*	*moderate*	low	low	low	low	*moderate*	*moderate*
	OS	serious	*moderate*	low	low	low	low	*moderate*	serious
Kulkarni, 2017	DSS	*moderate*	low	low	low	low	low	*moderate*	*moderate*
	OS	*moderate*	low	low	low	low	low	*moderate*	*moderate*
Nagao, 2017	DSS	*moderate*	*Ø* information	low	low	low	low	*moderate*	*moderate*
	OS	serious	*Ø* information	low	low	low	low	*moderate*	serious
*Population-based studies*									
Bekelman, 2013^1^	DSS	serious	*moderate*	low	low	low	low	*moderate*	serious
	OS	serious	*moderate*	low	low	low	low	*moderate*	serious
Bekelman, 2013^2^	DSS	*moderate*	*moderate*	low	low	low	low	*moderate*	*moderate*
	OS	*moderate*	*moderate*	low	low	low	low	*moderate*	*moderate*
Cahn, 2017	OS	**critical**	*moderate*	low	low	low	low	*moderate*	**critical**
Fischer-Valuck, 2018	OS	serious	*moderate*	low	low	low	low	*moderate*	serious
Ritch, 2018	OS	serious	*moderate*	low	low	low	low	*moderate*	serious
Seisen, 2017	OS	serious	*moderate*	low	low	low	low	*moderate*	serious
Smith, 2014	OS	serious	*moderate*	low	low	*moderate*	low	*moderate*	serious
Williams, 2018	DSS	*moderate*	*moderate*	low	low	low	low	*moderate*	*moderate*
	OS	serious	*moderate*	low	low	low	low	*moderate*	serious

low, Low RoB in this domain/overall; *moderate*, Moderate RoB in this domain/overall; serious, Serious RoB in this domain/overall; **critical,** Critical RoB in this domain/overall; *Ø* information, No information on which to base a judgement about RoB for this domain/overall.

DSS, disease-specific survival; OS, overall survival; RoB, risk of bias.

^1^Multivariable regression analysis, propensity score-adjusted regression analysis or inverse probability weighting-adjusted regression analysis.

^2^Instrumental variable analysis.

### Disease-specific survival

**Fig 2:**
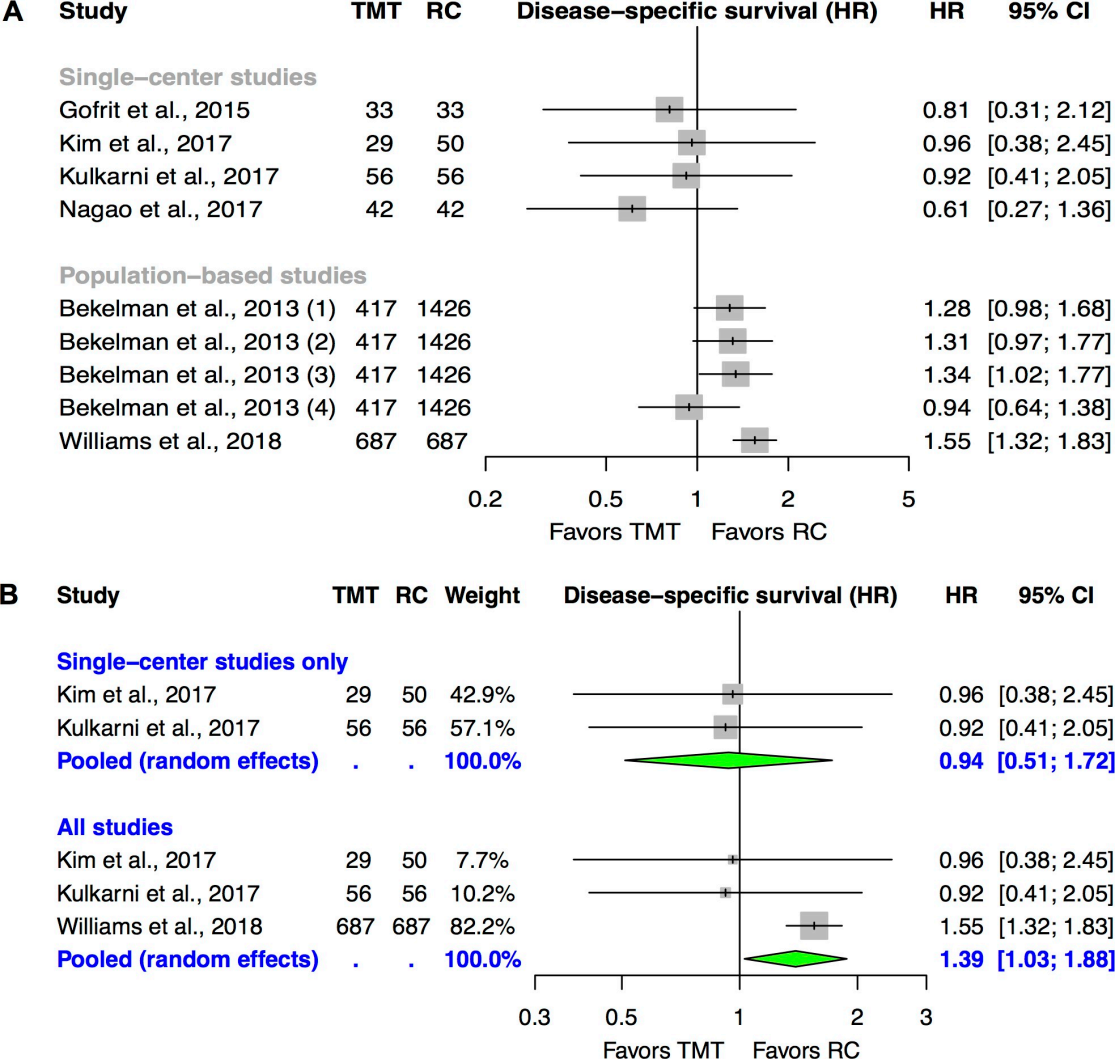
**Individual study results of studies reporting on disease-specific survival (A, N = 6) and the corresponding meta-analysis (B, N = 3).** The numbers in brackets next to the study names correspond to the numbers in *Table 2* and represent different analytic strategies. CIs in this figure might differ to the reported CIs in *Table 2* at the 2^nd^ decimal place due to imprecisions associated with log transformations. *CI*: *confidence interval; HR*: *hazard ratio; RC*: *radical cystectomy; TMT*: *trimodal therapy*.

The effect estimates and 95%-CIs of the identified studies that report on DSS (N = 6) are visually presented in the Forest plot of *Fig 2A*. The results of the single-center studies are all inconclusive with effect estimates in favor of TMT. Among the two population-based studies, *Bekelman et al*. reported one analytic strategy based on instrumental variable analysis (IVA) with a non-significant result (HR in favor of RC) and 3 non-IVA analytic strategies that were all significant or borderline significant (effect estimates in favor of RC). The recently published study of *Williams et al*. was the only identified investigation that reported on DSS and demonstrated clearly significant results in favor of RC (HR: 1.55 [95%-CI: 1.32–1.83]).

With regard to pooling of the single-center studies we *a prior*i excluded the investigations of *Gofrit et al*. and *Nagao et al*. due to “critical RoB” and due to potentially unreliable graphical effect size derivation in the presence of only a few outcome events, respectively. As both population-based studies relied on the same database and have a prominent overlap of 4 years, we selected *Williams et al*. for inclusion into the meta-analysis since this study in comparison to *Bekelman et al*. provided a more recent cohort and also used a comparable analytic strategy as *Kim et al*. and *Kulkarni et al*. Moreover, *Bekelman et al*. limited their study to cT2-T3 patients and all of their strategies except the IVA approach, which relies on strong assumptions, were at serious RoB. *Fig 2B* presents the pooled analysis within the strata “single-center studies only” (N = 2) and “all studies” (N = 3). The first analysis led to a non-significant summary measure of 0.94 (0.51–1.72), while the second one demonstrated a significant result in favor of RC (1.39 [1.03–1.88]). The latter result, however, did not statistically withstand sensitivity analyses in which the SEER-Medicare-based study of *Williams et al*. was iteratively replaced by the two analytic strategies of *Bekelman et al*. (IVA and propensity score-adjusted regression analysis, see *S5 Fig*).

### Overall survival

**Fig 3:**
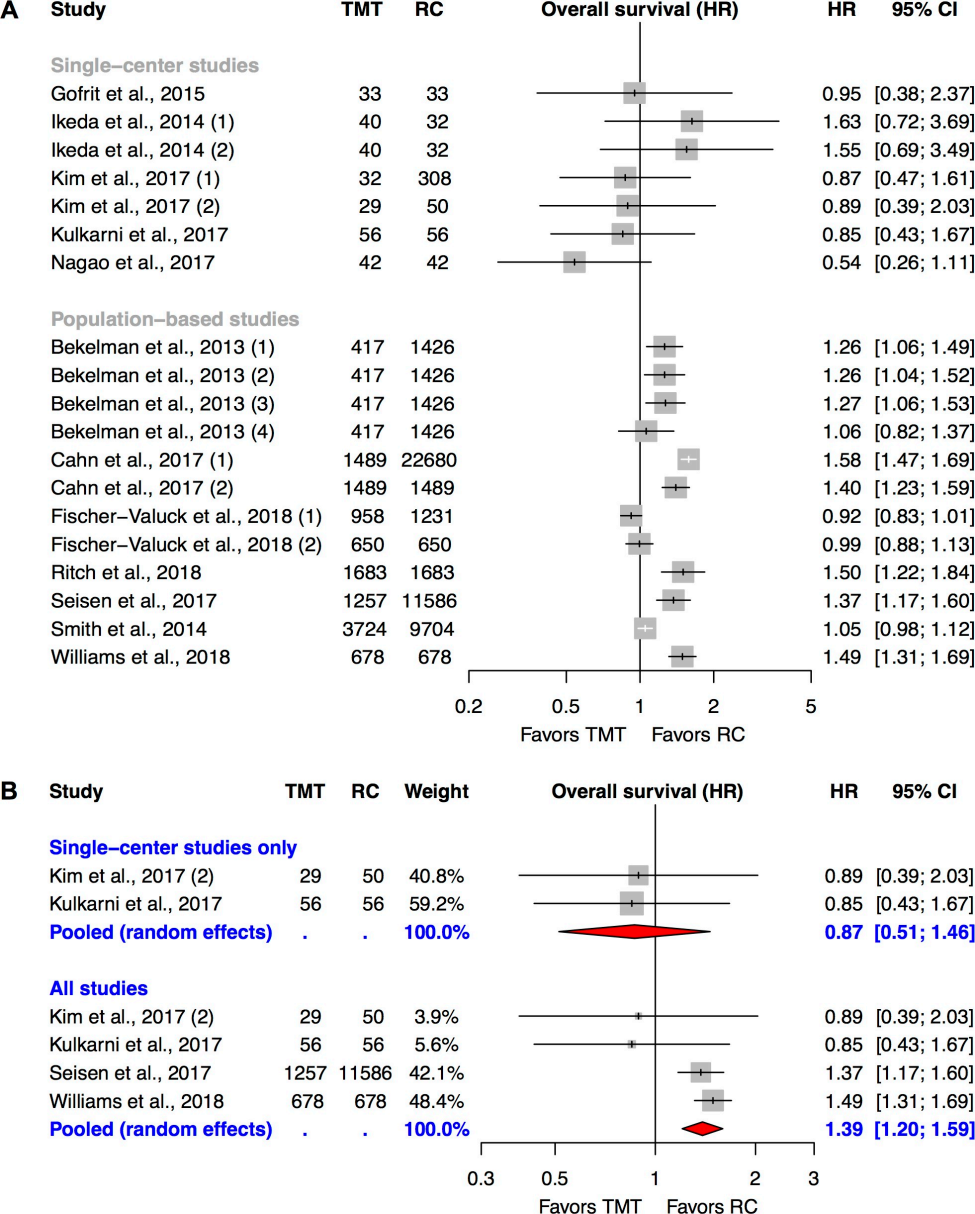
**Individual study results of studies reporting on overall survival (A, N = 12) and the corresponding meta-analysis (B, N = 4).** The numbers in brackets next to the study names correspond to the numbers in *Table 2* and represent different analytic strategies. CIs in this figure might differ to the reported CIs in *Table 2* at the 2^nd^ decimal place due to imprecisions associated with log transformations. *CI*: *confidence interval; HR*: *hazard ratio; RC*: *radical cystectomy; TMT*: *trimodal therapy*.

Twelve studies reported on OS and their effect sizes and 95%-CIs are presented in *Fig 3A* (stratified by single-center studies and population-based studies). As for DSS, all single-center studies (N = 5) provide inconclusive effect estimates with 95%-CIs that included the HR of 1. While all provided HRs in favor of TMT, the study of *Ikeda et al*., which was restricted to cT3-cT4 patients, reported effect estimates in favor of RC. Population-based studies relying on SEER-Medicare (*Bekelman et al*. and *Williams et al*.) demonstrated comparable patterns on the Forest plot as for DSS (see *Fig 2A*). The 5 population-based investigations from the NCDB displayed heterogeneity. *Cahn et al*., *Ritch et al*. and *Seisen et al*. who included cT2-T4 N0 M0 patients without any age restrictions estimated treatment effects significantly in favor of RC. *Fischer-Valuck et al*. and *Smith et al*. who focused on octogenarians and cT2 tumors, respectively, both reported non-significant treatment effects close to an HR of 1 with narrow 95%-CIs.

Only the study of *Kulkarni et al*. was rated as “moderate RoB” with regard to the outcome OS. Due to earlier described reasons we excluded the investigations of *Gofrit et al*. and *Nagao et al*. from the pooling of single-center studies reporting on OS. Furthermore, we decided to withdraw *Ikeda et al*. because of the exclusion of cT2 patients. The incorporation of population-based studies was limited to one SEER-Medicare-based investigation and one NCDB study, respectively. With regard to SEER-Medicare studies we preferred *Williams et al*. to *Bekelman et al*. as outlined earlier while we decided for the NCDB investigation of *Seisen et al*. The latter decision was based on the critical risk of bias assessment of *Cahn et al*., the age restrictions of *Fischer-Valuck et al*., the focus on cT2 tumors of *Smith et al*. and on the fact that *Seisen et al*. in comparison to *Ritch et al*. not only performed a sensitivity analysis for immortal time bias but also excluded non-urothelial histology.

The result of this meta-analysis is presented in *Fig 3B*. Pooling among single-center studies showed an inconclusive effect in favor of TMT (0.87 [0.51–1.46]) while the addition of the two selected population-based studies yielded an estimate statistically significantly in favor of RC (1.39 [1.20–1.59]). We performed several sensitivity analyses for the latter result in which we exchanged the study of *Seisen et al*. by the investigation of *Ritch et al*. and/or the work of *Williams et al*. by the study of *Bekelman et al*. (IVA approach and propensity score-adjusted regression analysis). All sensitivity analyses except the ones incorporating the IVA approach of *Bekelman et al*. were statistically robust (see *S6 Fig*).

## Discussion

This study systematically synthesized comparative evidence on DSS and OS for patients diagnosed with MIBC who were treated either by TMT or RC. Three out of 6 eligible studies that report on DSS were considered “moderate RoB” and qualified for quantitative analysis that demonstrated a result in favor of RC (1.39 [1.03–1.88]). Among 12 eligible studies providing effect estimates with regard to OS, 4 investigations could be included into a meta-analysis rendering a pooled estimate clearly in favor of RC (1.39 [1.20–1.59]). However, 3 out of the 4 studies incorporated into this quantitative synthesis were rated as “serious RoB”. Studies focusing on cT2 tumors or on older patients with higher competing risks demonstrated inconclusive results on OS. For both outcomes DSS and OS, we detected comparable patterns in the body of evidence which consisted on one hand of small-sample single-center studies with wide 95%-CIs and HRs close to 1 or slightly in favor of TMT and on the other hand of United States-based research at the population level with narrow 95%-CIs and effect estimates that were preponderantly in favor of RC.

There are several explanations for the heterogeneity in results between single-center studies and population-based studies. First, TMT is a complex treatment modality that requires a highly specialized and multidisciplinary provider team to select ideal patients, perform a maximal TURBT, safely apply chemoradiation, perform cystoscopic follow-up examinations in bladders heavily altered by postradiation changes and to rigorously recommend and perform salvage RC in the event of treatment failure. While the single-center studies provide evidence from such specialized provider teams (efficacy), the population-based studies rather report more generalizable, population-wide and real-world estimates (effectiveness). It is generally known that efficacy results are more in favor of an intervention (i.e. in this case, TMT) than effectiveness results. Second, platinum-based concurrent chemotherapy as well as salvage RC are integral components of true TMT. Therefore, the circumstance that the NCBD studies could not determine the receipt of platinum-based therapy and the obviously different salvage RC rates between single-center studies and population-based studies (11% in *Kulkarni et al*. [23] versus 2% in *Ritch et al*. [27]) might also partially explain the heterogeneity.

Third, the exposure (TMT versus RC)–outcome (DSS, OS) relationship is moderately (DSS) and strongly (OS) confounded by the comorbidity/performance of the patient and even sophisticated analytic strategies as utilized in certain studies cannot exclude unmeasured confounding. Reliable measurement of relevant confounding domains is thus crucial to address confounding bias as much as possible. All population-based studies used the Charlson Comorbidity Index to address the confounding theme “comorbidity/performance”. *Bekelman et al*. [24] performed in addition an IVA which theoretically accounts for both measured and unmeasured confounding [31]. Although such a methodology relies on strong theoretical assumptions, they could demonstrate a change of the effect estimate in the direction of the null hypothesis. Hence, a larger amount of unmeasured confounding in the population-based studies because of a lack of performance status measures could be another explanation for the heterogeneity in results between single-center studies and population-based studies.

The strengths of our approach are as follows: First, we performed a comprehensive and rigorous RoB assessment using the ROBINS-I instrument. Prior published systematic reviews and meta-analyses on the same research question either used the Newcastle-Ottawa Scale (NOS) [11] / Methodological Index for Non-Randomized Studies (MINORS) [10] for RoB assessment or did not perform a RoB assessment [9]. We strongly believe that a detailed RoB assessment in this setting is not only highly warranted but also that the ROBINS-I instrument is by design superior to the NOS/MINORS tools when it comes to the evaluation of confounding bias, the most important bias influencing our research question. Second, our meta-analysis involved, according to best practice, pooling of sufficiently adjusted HRs. Prior meta-analyses either pooled unadjusted HRs of comparative studies [11], quantitatively synthesized studies that are at diametral different RoB [10] or simply compared pooled survival estimates of separate TMT series and RC series with a t-test [9], all of which are severely biased strategies to draw causal inference for the current research question due to confounding, heterogeneity and ecological bias, respectively. Third, in the presence of experimental evidence favoring concurrent chemotherapy during RT to RT alone [7] we only included TMT arms in which a majority of patients received concurrent chemotherapy; prior systematic reviews and meta-analyses included a mix of bimodal and trimodal therapy [9–11]. Fourth and finally, this work represents updated systematic summary of comparative evidence of TMT versus RC for MIBC (last search update: August 1, 2018). Such an updated search was highly warranted as 8 out of 12 identified studies were published in 2017 or 2018 and prior evidence syntheses utilized outdated searches from 2013 [9] and 2016 [11].

However, this evidence synthesis is not without limitations. First, the overall quality of evidence is limited by the observational nature of the included studies. Therefore, this investigation is still biased by unmeasured confounding and cannot provide better quality of evidence than “moderate RoB” and “serious RoB” for DSS and OS, respectively, Second, all population-based studies originated from two United States-based databases and suffer not only from partial overlap between the databases but also from significant overlap within each database. Thus, we were only able to include two population-based studies from two different databases into quantitative synthesis. Since the decisions to include/exclude such studies were prone to some subjectivity, we tried to attenuate this by using as explicit and objective selection criteria as possible and by performing sensitivity analyses. Unfortunately, most of the sensitivity analyses were highly influential as each population-based study included a high number of patients. Third, thorough RoB assessment is driven by the quality and quantity of the provided study information. The latter was often a limiting factor in the absence of pre-registered protocols. However, we tried to mitigate this limitation by contacting study authors if relevant details were lacking. Fourth and finally, several studies were designed and conducted according to our eligibility criteria but, unfortunately, analyzed and presented in an inefficient way that does not allow for drawing causal inference.

The findings of this systematic review and meta-analysis are summarized in the format of the GRADE evidence profile (see *S2 Table*). Based on this methodology, the certainty of evidence for both outcomes DSS and OS was rated as “very low” due to the RoB and the inconsistency of results. Thus, based on the available evidence at this time, the choice between TMT or RC for MIBC depends on individual patient preferences, the recommendation of a multidisciplinary provider team experienced with both approaches and consideration of immediate surgical mortality associated with RC versus hypothetically worse oncological long-term outcomes related to TMT.

We highly expect that further research will have an important impact on the confidence in the estimate of the treatment effect. As we do not expect landmark results from experimental research within the near future [8], the arrival of further observational comparative research has to be awaited. As a lesson learned from this systematic review and meta-analysis, such investigations have to fulfil certain requirements such as rigorous adjustment for confounding bias (including the incorporation of comorbidity indexes and performance measures), meaningful subgroup analyses (such as by age and clinical T stage), full methodological and analytical transparency (including use of online appendices in light of strict word count limitations), strict adherence to reporting guidelines (STROBE Statement (Strengthening the Reporting of Observational Studies in Epidemiology) [32]) and, if population-based, incorporation of non-United States jurisdictions.

## Conclusions

TMT is an alternative to RC for MIBC, especially in patients with a high operative risk and in those not willing to sacrifice their bladder. This study systematically synthesized the currently available observational comparative evidence both qualitatively and quantitatively. Pooled results were significant in favor of RC (DSS: moderate RoB, OS: serious RoB). However, the favorability of RC is mainly driven by the large population-based studies that are at high risk for confounding or information bias. Therefore, the certainty of these treatment estimates can be considered very low and further research will likely have an important impact on these estimates. As no randomized evidence that would ultimately state superiority/non-inferiority of one of the two modalities in a confounding-free setting is expected in the near future, high-quality comparative studies thoroughly adjusting for tumor characteristics and comorbidities/performance are warranted to guide clinical decision-making in the meantime.

## Supporting information

S1 Appendix PRISMA checklist. (PDF)

S1 Fig Continuous-course versus split-course trimodal therapy. (TIFF)

S2 Fig Causal diagram of the exposure (TMT versus RC)–outcome (DSS, OS) relationship (A: Unadjusted, B: Adjusted for the themes “Tumor-specific factors” and “Comorbidity/performance”). Green arrows represent causal pathways of interest while red and black arrows represent biasing and blocked/adjusted pathways, respectively. *DSS*: *disease-specific survival; OS*: *overall survival; RC*: *radical cystectomy*, *TMT*: *trimodal therapy;*
(TIFF)

S3 Fig Matrix demonstrating for each included study if controlling for certain variables was performed or not. (TIF)

S4 Fig Definitions of survival time among population-based studies and resulting biases. (TIFF)

S5 Fig Sensitivity analyses related to the pooling of studies reporting on disease-specific survival. The numbers in brackets next to the study names correspond to the numbers in *Table 2* and represent different analytic strategies. CIs in this figure might differ to the reported CIs in *Table 2* at the 2nd decimal place due to imprecisions associated with log transformations. *CI*: *confidence interval; HR*: *hazard ratio; RC*: *radical cystectomy; TMT*: *trimodal therapy;*
(TIFF)

S6 Fig Sensitivity analyses related to the pooling of studies reporting on overall survival. The numbers in brackets next to the study names correspond to the numbers in *Table 2* and represent different analytic strategies. CIs in this figure might differ to the reported CIs in *Table 2* at the 2nd decimal place due to imprecisions associated with log transformations. *CI*: *confidence interval; HR*: *hazard ratio; RC*: *radical cystectomy; TMT*: *trimodal therapy;*.
(TIFF)

S1 Table Search strategy. (PDF)

S2 Table GRADE evidence profile of the question “Should trimodal therapy versus radical cystectomy be used for muscle-invasive bladder cancer?”. (PDF)

S1 Text Supplemental methods. (PDF)

S2 Text Supplemental results. (PDF)
