# Supplementary figures and images for: Systematic review and meta-analysis on trimodal therapy versus radical cystectomy for muscle-invasive bladder cancer: Does the current quality of evidence justify definitive conclusions?
Source: PLoS One. 2019 Apr 29;14(4):e0216255. doi: 10.1371/journal.pone.0216255 (PMC6488073; doi:10.1371/journal.pone.0216255)

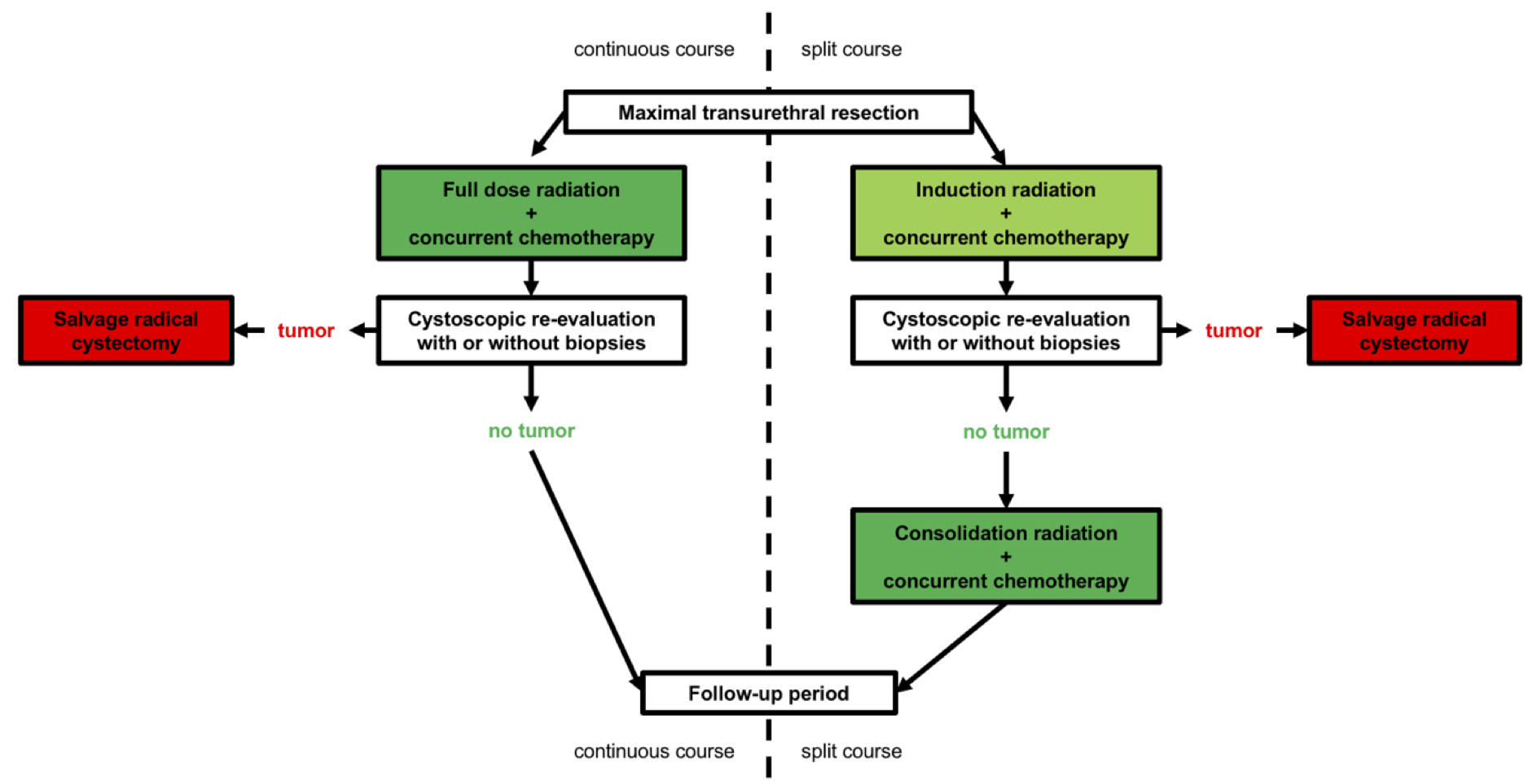

Supplement: S1 Fig — (TIFF) [file pone.0216255.s002.tiff]

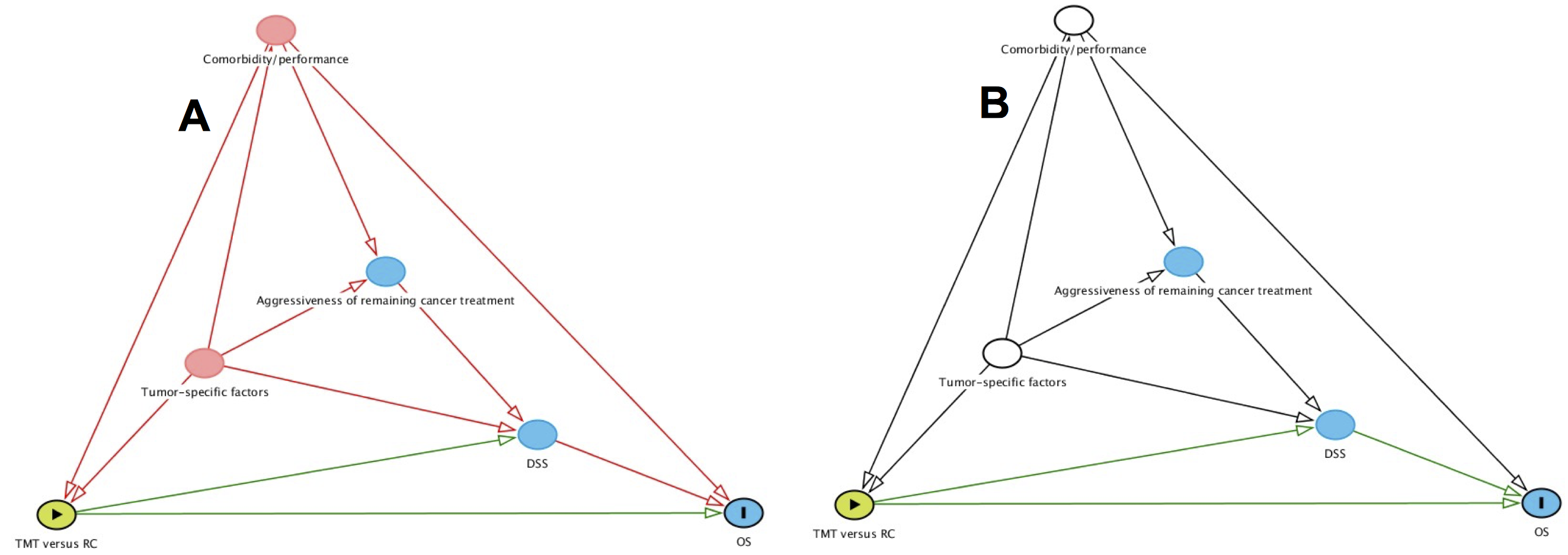

Supplement: S2 Fig — Green arrows represent causal pathways of interest while red and black arrows represent biasing and blocked/adjusted pathways, respectively. DSS: disease-specific survival; OS: overall survival; RC: radical cystectomy, TMT: trimodal therapy; (TIFF) [file pone.0216255.s003.tiff]

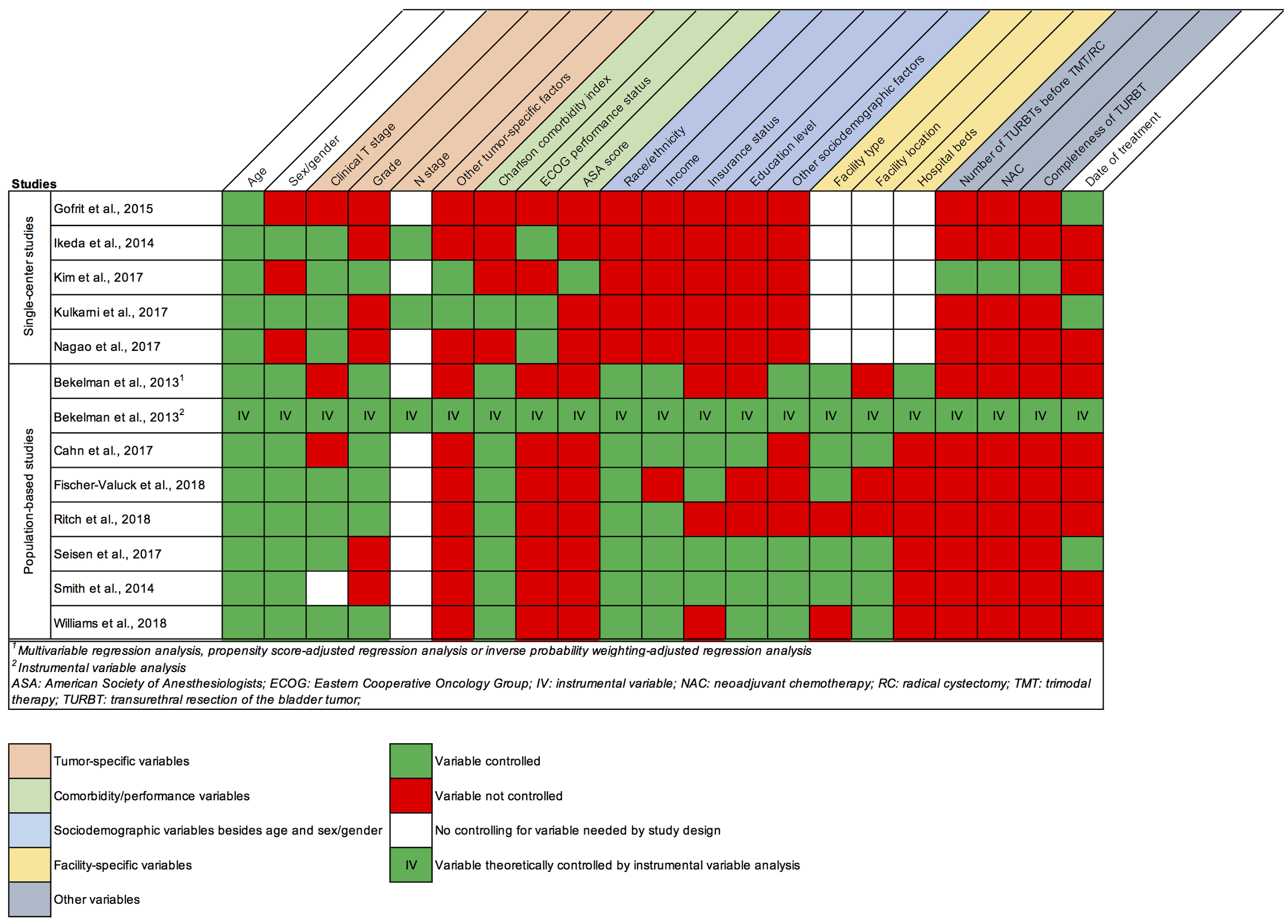

Supplement: S3 Fig — (TIF) [file pone.0216255.s004.tif]

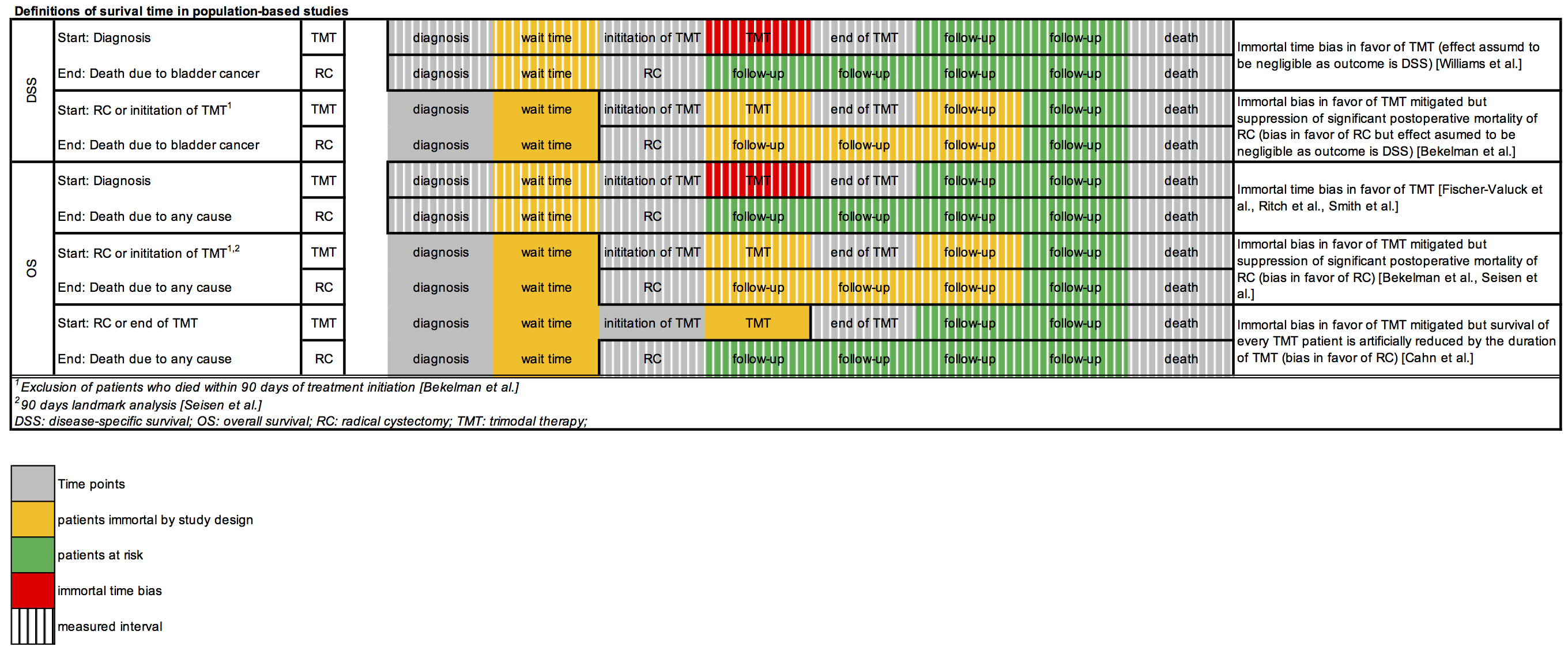

Supplement: S4 Fig — (TIFF) [file pone.0216255.s005.tiff]

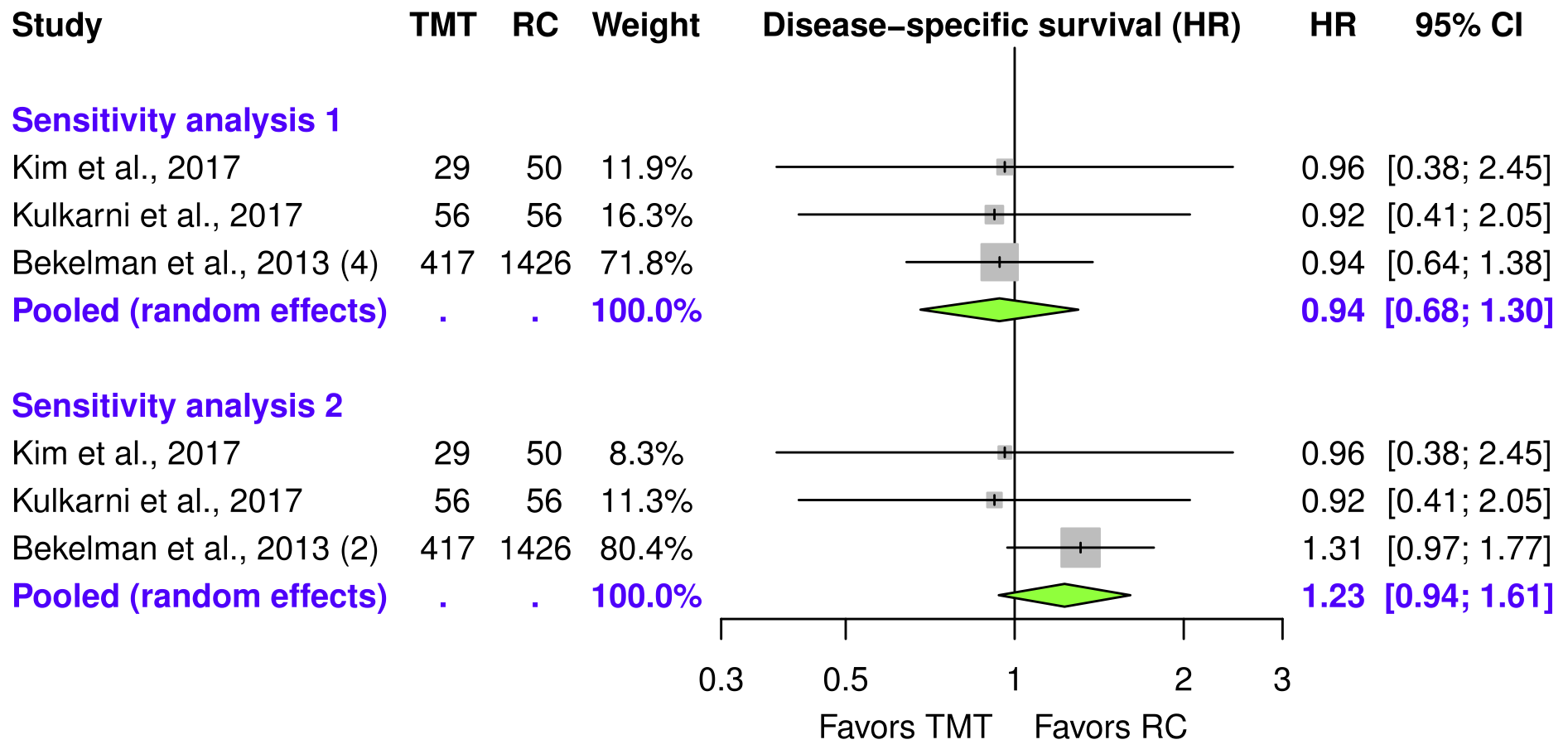

Supplement: S5 Fig — The numbers in brackets next to the study names correspond to the numbers in Table 2 and represent different analytic strategies. CIs in this figure might differ to the reported CIs in Table 2 at the 2nd decimal place due to imprecisions associated with log transformations. CI: confidence interval; HR: hazard ratio; RC: radical cystectomy; TMT: trimodal therapy; (TIFF) [file pone.0216255.s006.tiff]

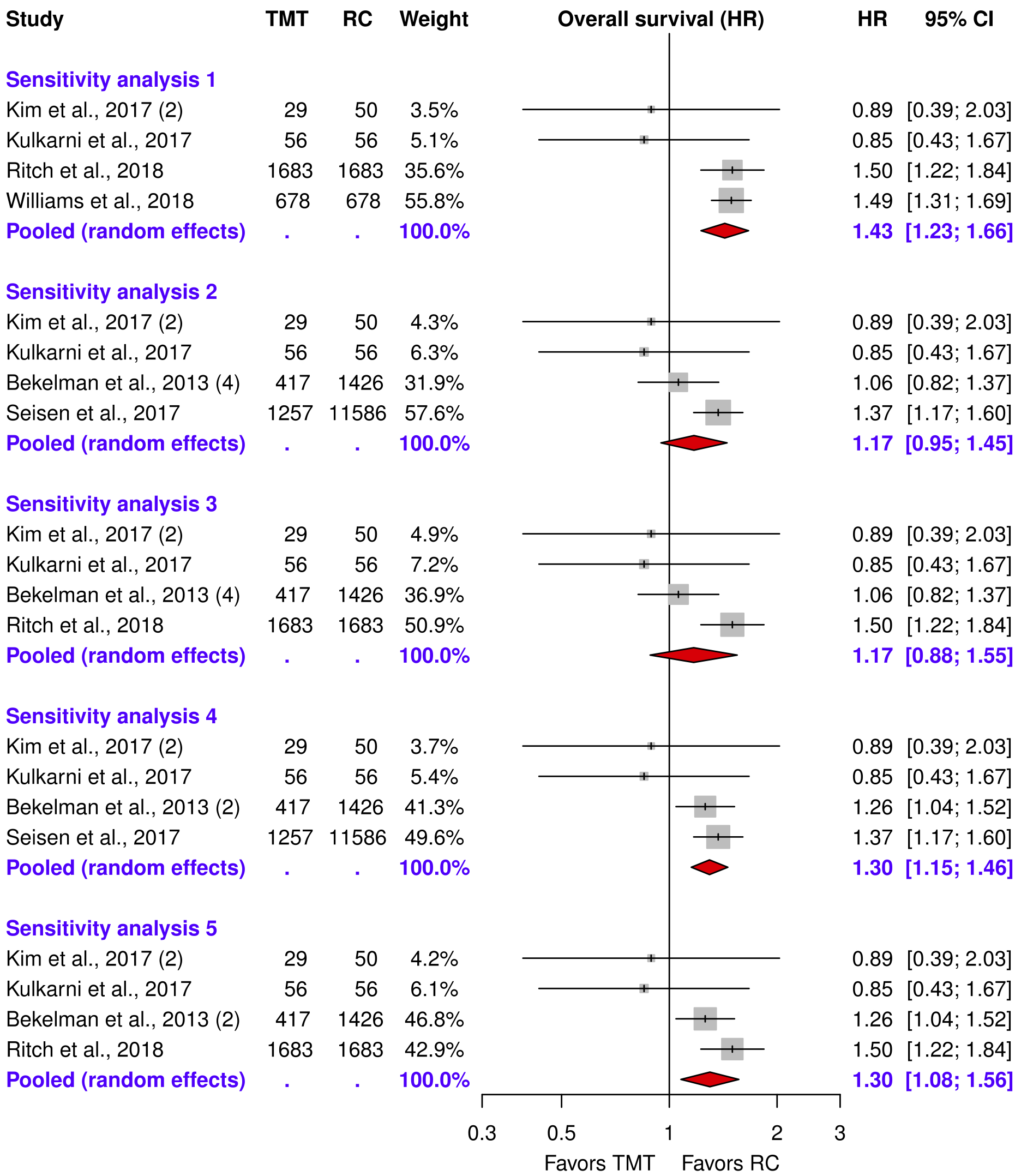

Supplement: S6 Fig — The numbers in brackets next to the study names correspond to the numbers in Table 2 and represent different analytic strategies. CIs in this figure might differ to the reported CIs in Table 2 at the 2nd decimal place due to imprecisions associated with log transformations. CI: confidence interval; HR: hazard ratio; RC: radical cystectomy; TMT: trimodal therapy;. (TIFF) [file pone.0216255.s007.tiff]
