# Supplementary material for: Systematic review and meta-analysis on trimodal therapy versus radical cystectomy for muscle-invasive bladder cancer: Does the current quality of evidence justify definitive conclusions?
Source: PLoS One. 2019 Apr 29;14(4):e0216255. doi: 10.1371/journal.pone.0216255 (PMC6488073; doi:10.1371/journal.pone.0216255)
Supplement: S1 Table — (PDF) [file pone.0216255.s008.pdf]

Supplemental Table 1. Search strategy.

MEDLINE:
The search strategy for OvidSP MEDLINE (1946 to **January Week 5 2018**) retrieved **560** references of which **558** were unique and not duplicated in our other searches. I used a combination of MeSH and free text terms for

#	Searches	Results	Comment
1	((transurethral adj3 resect*) or "tur-bladder" or turb or turbt or TmLRBT).ti,ab,kf.	9699	Transurethral resection terms
2	exp Antineoplastic Agents/ or (antineoplastic adj2 agent*).mp.	968748	Chemotherapy drugs
3	combined modality therapy/ or chemoradiotherapy/ or chemoradiotherapy, adjuvant/ or chemotherapy, adjuvant/ or neoadjuvant therapy/ or photochemotherapy/ or radioimmunotherapy/ or radiotherapy, adjuvant/ or ((combined adj2 modality) or (adjuvant adj2 chemotherap*) or chemoradiotherap* or photochemotherap* or radioimmunotherap*).ti,ab,kf.	244688	
4	Organ Sparing Treatments/ or (organ adj2 sparing).ti,ab,kf. [****MeSH since 2012****]	2992	
5	Antineoplastic protocols/ or Antineoplastic Combined Chemotherapy Protocols/ or ((anticancer* or antineoplas*) adj5 (agent* or protocol* or regimen*)).ti,ab,kf.	149043	
6	or/3-5	353024	Combined modality therapies
7	Urinary Bladder Neoplasms/ or (bladder adj2 (cancer* or neoplas* or tumor* or tumour*)).ti,ab,kf.	54355	
8	Carcinoma, Transitional Cell/	17399	
9	((transitional adj2 cell adj2 carcinoma*) or (urothelial adj2 carcinom*)).ti,ab,kf.	14534	
10	or/7-9	60324	Urinary Bladder cancer terms
11	1 and 2 and 6 and 10	560	Base clinical set results

Medline -in-Process & Other Non-Indexed Citations

The search strategy for OvidSP **M**edline -in-Process & Other Non-Indexed Citations February 9, 2018> retrieved **20** references of which **7** were unique and not duplicated in our other searches. I used a combination of free text terms for

#	Searches	Results	Comment
1	((transurethral adj3 resect*) or "tur-bladder" or turb or turbt or TmLRBT).ti,ab,kf.	1116	Transurethral resection terms
2	((antineoplastic adj2 (drug* or agent*)) or (cisplatin or "mitomycin c")).mp.	5939	Chemotherapy drugs
3	((combined adj2 modality) or ((neoadjuvant or adjuvant or cancer) adj2 chemotherap*) or chemoradiotherap* or photochemotherap* or radioimmunotherap*).ti,ab,kf.	7697	
4	(organ adj2 sparing).ti,ab,kf.	96	
5	((anticancer* or antineoplas*) adj5 (agent* or protocol* or regimen*)).ti,ab,kf.	2126	
6	or/3-5	9776	Combined modality therapies
7	(bladder adj2 (cancer* or neoplas* or tumor* or tumour*)).ti,ab,kf.	3631	
8	((transitional adj2 cell adj2 carcinoma*) or (urothelial adj2 carcinom*)).ti,ab,kf.	1649	
9	or/7-8	4581	Urinary Bladder cancer terms
10	1 and 2 and 6 and 9	20	Base clinical set results

MEDLINE(R) Epub Ahead of Print
The search strategy for OvidSP Medline Epub Ahead of Print, February 9, 2018 retrieved **1** references of which **1** were unique and not duplicated in our other searches. I used a combination of free text terms for

#	Searches	Results	Comment
1	((transurethral adj3 resect*) or "tur-bladder" or turb or turbt or TmLRBT).ti,ab,kf.	137	Transurethral resection terms
2	((antineoplastic adj2 (drug* or agent*)) or (cisplatin or "mitomycin c")).mp.	1238	Chemotherapy drugs
3	((combined adj2 modality) or ((neoadjuvant or adjuvant or cancer) adj2 chemotherap*) or chemoradiotherap* or photochemotherap* or radioimmunotherap*).ti,ab,kf.	1641	
4	(organ adj2 sparing).ti,ab,kf.	20	
5	((anticancer* or antineoplas*) adj5 (agent* or protocol* or regimen*)).ti,ab,kf.	562	
6	or/3-5	2190	Combined modality therapies
7	(bladder adj2 (cancer* or neoplas* or tumor* or tumour*)).ti,ab,kf.	703	
8	((transitional adj2 cell adj2 carcinoma*) or (urothelial adj2 carcinom*)).ti,ab,kf.	301	
9	or/7-8	859	Urinary Bladder cancer terms
10	1 and 2 and 6 and 9	1	Base clinical set results

EMBASE
The search strategy for OvidSP Embase Classic+Embase <1947 to 2018 February 9, 2018> retrieved **1600** references of which **1251** were unique and not duplicated in our other searches. I used a combination of EMBASE and free text terms for

#	Searches	Results	Comment
1	transurethral resection/	18194	
2	((transurethral adj3 resect*) or "tur-bladder" or turb or turbt or TmLRBT).ti,ab.	16062	
3	or/1-2	23312	Transurethral resection terms
4	exp antineoplastic agent/	1990641	
5	(antineoplastic adj2 agent*).mp.	267692	
6	cancer chemotherapy/	207094	
7	or/4-6	2029961	Chemotherapy drugs
8	multimodality cancer therapy/	65030	
9	combination chemotherapy/ or cancer combination chemotherapy/	119030	
10	adjuvant chemotherapy/ or adjuvant chemoradiotherapy/	42214	
11	adjuvant radiotherapy/ or cancer radiotherapy/ or adjuvant chemoradiotherapy/ or chemoradiotherapy/	187735	
12	cancer adjuvant therapy/ or adjuvant therapy/ or cancer therapy/	179157	
13	cancer immunotherapy/	46042	
14	cancer radiotherapy/	158393	
15	intensity modulated radiation therapy/	23197	
16	external beam radiotherapy/ or conformal radiotherapy/	21360	
17	((combined adj2 modality) or (adjuvant adj2 chemotherap*) or chemoradiotherap* or photochemotherap* or radioimmunotherap* or (modulated adj2 therap*)).ti,ab.	80131	
18	conservative treatment/ [****Equivalent to MeSh organ sparing treatments****]	72404	
19	bladder sparing trimodal therapy/	1	
20	clinical protocol/	84487	
21	((anticancer* or antineoplas*) adj5 (agent* or protocol* or regimen*)).ti,ab.	29508	
22	or/8-21	752070	Combined modality therapies
23	muscle invasive bladder cancer/	2088	Muscle invasive term
24	3 and 7 and 22 and 23	213	base clinical set 1
25	bladder tumor/ or urinary tract tumor/	21439	
26	bladder cancer/ or bladder carcinogenesis/ or bladder carcinoma/ or bladder metastasis/	57746	
27	transitional cell carcinoma/	23435	
28	((transitional adj2 cell adj2 carcinoma*) or (urothelial adj2 carcinom*)).ti,ab.	22865	
29	(bladder adj2 (cancer* or neoplas* or tumor* or tumour*)).ti,ab.	50410	
30	or/25-29 [***urinary bladder cancer terms****]	97308	Urinary Bladder cancer terms
31	3 and 7 and 22 and 30	1597	Base clinical set 2
32	24 or 31	1600	Final results

Cochrane

The search strategy for Cochrane issue 1 or 12, January 2018> retrieved **85** references of which **20** were unique and not duplicated in our other searches. I used a combination of MeSH and free text terms for

Search Name:
Date Run: 11/02/18 16:15:48.86
Description:

ID	Search	Hits	Comment
#1	(transurethral near/3 resect*) or "tur-bladder" or turb or turbt or TmLRBT	1910	Transurethral resection terms
#2	MeSH descriptor: [Antineoplastic Agents] explode all trees	12625	
#3	(antineoplastic near/2 agent*)	12610	
#4	[or #2-#3]	15413	Chemotherapy drugs
#5	MeSH descriptor: [Combined Modality Therapy] this term only	14704	
#6	MeSH descriptor: [Chemoradiotherapy] this term only	580	
#7	MeSH descriptor: [Chemoradiotherapy, Adjuvant] this term only	145	
#8	MeSH descriptor: [Chemotherapy, Adjuvant] this term only	3942	
#9	MeSH descriptor: [Neoadjuvant Therapy] this term only	1078	
#10	MeSH descriptor: [Photochemotherapy] this term only	848	
#11	MeSH descriptor: [Radioimmunotherapy] this term only	59	
#12	MeSH descriptor: [Radiotherapy, Adjuvant] this term only	1052	
#13	(combined near/2 modality) or (adjuvant near/2 chemotherap*) or chemoradiotherap* or photochemotherap* or radioimmunotherap*	26736	
#14	MeSH descriptor: [Organ Sparing Treatments] this term only	112	
#15	(organ near/2 sparing)	145	
#16	MeSH descriptor: [Antineoplastic Protocols] this term only	28	
#17	MeSH descriptor: [Antineoplastic Combined Chemotherapy Protocols] this term only	13314	
#18	((anticancer* or antineoplas*) near/5 (agent* or protocol* or regimen*))	24309	
#19	[or #5-#18]	45032	Combined modality therapies
#20	MeSH descriptor: [Urinary Bladder Neoplasms] this term only	1234	
#21	(bladder near/2 (cancer* or neoplas* or tumor* or tumour*))	2727	
#22	MeSH descriptor: [Carcinoma, Transitional Cell] this term only	455	
#23	(transitional NEAR/j2 cell near/2 carcinoma*) or (urothelial near/2 carcinom*)	333	
#24	[or #20-#23]	2898	Urinary Bladder cancer terms
#25	#1 and #4 and #19 and #24	85	Base clinical set results
