# Supplementary material for: Systematic review and meta-analysis on trimodal therapy versus radical cystectomy for muscle-invasive bladder cancer: Does the current quality of evidence justify definitive conclusions?
Source: PLoS One. 2019 Apr 29;14(4):e0216255. doi: 10.1371/journal.pone.0216255 (PMC6488073; doi:10.1371/journal.pone.0216255)
Supplement: S2 Table — (PDF) [file pone.0216255.s009.pdf]

Supplemental Table 2. GRADE evidence profile of the question "Should trimodal therapy versus radical cystectomy be used for muscle-invasive bladder cancer?"

Certainty assessment							Impact (HR	Certainty	Importance
№ of studies	Study design	Risk of bias	Inconsistency	Indirectness	Imprecision	Other considerations			
Disease-specific survival (follow up: range 31 months to 54 months)									
3	observational studies	serious [a]	serious [b,c]	not serious	not serious	none	HR: 1.39 (95%-CI: 1.03 to 1.88) [№ (TMT): 763, № (RC): 793] [d]	⊕◯◯◯ VERY LOW	CRITICAL
Overall survival (follow up: range 20 months to 54 months)									
4	observational studies	very serious [e]	serious [b,c]	not serious	not serious	none	HR: 1.39 (95%-CI: 1.20 to 1.59) [№ (TMT): 2020, № (RC): 12379] [d]	⊕◯◯◯ VERY LOW	CRITICAL

CI: Confidence interval; **HR:** Hazard Ratio
a. all studies included in meta-analysis were rated as "moderate risk of bias" (ROBINS-I)
b. inconsistency between single-center studies and population-based studies
c. conclusion not robust to sensitivity analyses
d. TMT: trimodal therapy; RC: radical cystectomy
e. 3/4 studies included in meta-analysis were rated as "serious risk of bias" (ROBINS-I)
