# Supplementary material for: Systematic review and meta-analysis on trimodal therapy versus radical cystectomy for muscle-invasive bladder cancer: Does the current quality of evidence justify definitive conclusions?
Source: PLoS One. 2019 Apr 29;14(4):e0216255. doi: 10.1371/journal.pone.0216255 (PMC6488073; doi:10.1371/journal.pone.0216255)
Supplement: S1 Text — (PDF) [file pone.0216255.s010.pdf]

S1 Text. Supplemental methods.

Eligible Studies

Non-randomized studies included quasi-randomized controlled trials, non-randomized controlled trials, prospective cohort studies, retrospective cohort studies and historically controlled trials. Controlled before-and-after studies, interrupted-time-series analyses, cross-sectional studies, case series, case reports, economic studies, decision analyses, review articles, commentaries and editorials were excluded. Furthermore, works not subject to peer-review including theses/dissertations, reports from governmental agencies and conference abstracts were not considered.

Search Strategy, Study Selection, Data Collection and Data Items

The final search strategy as presented in *Supplemental Table 1* was developed in an iterative process. First, our review team drafted a search framework that was translated into a preliminary strategy by a health science librarian with extensive expertise in systematic reviews (EMU). Second, the preliminary search strategy was piloted to allow the assessment of its sensitivity and of potential patterns of missingness. Third and finally, the results of the preliminary screening were used to refine the preliminary search strategy. In addition, literature saturation was ascertained by screening of the reference lists of relevant original articles, systematic and narrative reviews identified during the search process. In case the systematic literature search yielded records with eligible English abstracts but full-text articles in other languages, we tried to retrieve and translate the full-text article with the help of bi-/multilingual personal within our institution. Data management of all study records retrieved during the systematic search process was carried out by DistillerSR (Evidence Partners, Ottawa, Canada) to allow for consistency checks and auditing at all levels of the review. A multilevel study selection process (title screening, abstract screening, full-text screening) and a hierarchical data extraction process were performed by two independent reviewers (MSW, JKR, CP). In case of persisting disagreement after discussion, a third reviewer was consulted (MSW, CJD, ZK or GSK). The study selection and the data extraction forms were piloted at all levels with 20 references from the preliminary search and refined accordingly. Furthermore, all reviewers involved in the two processes took part in calibration and training exercises. For each included study, data on the design (temporality, type, location, N_{total}, accrual period, follow-up time), on the population (T stage, N stage, M stage, other eligibility criteria), on trimodal therapy (TMT; N_{TMT}, continuous versus split course, dose of radiation therapy, concurrent chemotherapy, neoadjuvant or adjuvant therapies), on radical cystectomy (RC; N_{RC}, extent of pelvic lymphadenectomy, neoadjuvant or adjuvant therapies) and on the analytic strategy were abstracted.

Risk of Bias Assessment

By nature of the research question, the derived eligibility criteria and the framework of the ROBINS-I tool (Risk Of Bias In Non-randomized Studies – of Interventions), "confounding" was the most critical risk of bias (RoB) domain dominating the overall RoB assessment per study. We assumed that the exposure (trimodal therapy versus radical cystectomy) – outcome (disease-specific survival, overall survival) relationship is predominantly confounded by the themes "tumor-specific factors" and "comorbidity/performance". Therefore, the assessment "moderate RoB" was only assigned if a study controlled for the two themes by reliable variables. Namely, "tumor-specific factors" had to be reliably addressed by a variable for the clinical T stage variable (plus cN stage variable if N+ patients were included) while "comorbidity/performance" had to be controlled by a comorbidity and (OS) / or (DSS) a performance measure. Failure to fulfill these requirements and/or the incorporation of adjustment factors considered being a part of the causal chain led to assessments "serious RoB" or "critical RoB" depending on the severity of the violations.
