# Supplementary material for: Systematic review and meta-analysis on trimodal therapy versus radical cystectomy for muscle-invasive bladder cancer: Does the current quality of evidence justify definitive conclusions?
Source: PLoS One. 2019 Apr 29;14(4):e0216255. doi: 10.1371/journal.pone.0216255 (PMC6488073; doi:10.1371/journal.pone.0216255)
Supplement: S2 Text — (PDF) [file pone.0216255.s011.pdf]

S2 Text. Supplemental results.

Study Selection

A total of 12 studies were eligible for inclusion into qualitative synthesis. The database search yielded 2372 records while 13 records were identified through other sources (hand search: 3; screening of reference lists of older systematic and narrative review articles: 10). A total of 1934 unique records were screened after deduplication of which 258 qualified for full-text analysis (kappa statistic at title screening (exclusion versus inclusion/unclear, reviewer 1 versus reviewer 2): 0.58, kappa statistic at abstract screening (exclusion versus inclusion/unclear, reviewer 1 versus reviewer 2): 1.00). The electronic full text was not retrievable for one record in Chinese language. Of the remaining 257 articles, 172 consisted of a non-comparative design and/or involved heterogeneous treatment regiments which did not allow the identification of an eligible trimodal therapy arm. Sixty-seven were considered as conference abstracts, 4 did not allow the derivation of treatment effects due to unhelpful analytic strategies and 2 articles were editorials.

Risk of Bias Assessment

The evaluation of the domain "confounding" was strictly based on the matrix in *Supplemental Figure 3* that graphically demonstrates for each included study if controlling for certain variables was performed or not. As the study of *Bekelman et al.* [24] presented instrumental variable analysis (IVA) as one of the four analytic strategies which theoretically controls for both measured and unmeasured confounding [31], we rated the domain "confounding" in their study twice. The non-IVA approaches were rated as "serious risk of bias (RoB)" while the IVA approach was graded as "moderate RoB". Under the assumption of the first judgment ("moderate RoB") on *Bekelman et al.* [24], 4 out of 6 studies that report on disease-specific survival (DSS) and only 1 out of 11 studies that report on overall survival (OS) were rated as "moderate RoB" in the domain "confounding". The remaining studies/outcomes were all rated as "serious RoB" except *Gofrit et al.* (hard-matched on age and year of treatment) [19] and *Cahn et al.* (use of pathologic instead of clinical T stage as adjustment factor) [30] that both received the judgment "critical RoB" with regard to this domain. The other RoB domains were almost exclusively rated as "moderate RoB" (selection of the reported results) or "low RoB" (remaining domains) since (1) the studies did not selectively exclude patients after the application of trimodal therapy (TMT) / radical cystectomy (RC), (2) TMT and RC are clearly identifiable interventions with a negligible risk of deviation after initiation, (3) missing data are effectively addressed by the time-to-event analysis, (4) death events as outcomes are relatively robust to measurement bias and (5) the studies were consistent with an *a priori* plan in the absence of a pre-registered protocol. The domain "selection of participants" was rated as "moderate RoB" in all population-based studies as they are all exposed to a certain amount of selection bias in favor of TMT or RC by the way they defined survival time (see *Supplemental Figure 4* for more details). Moreover, 5 out of the 7 population-based studies [24-26,29,31] even introduced further selection bias as they were restricted by design to *continuous-course* regimens and *split-course* regimens with complete response at re-evaluation. Hence, *split-course* TMT patients who do not manifest a complete response at re-evaluation and consecutively undergo salvage RC were systematically excluded from the respective study populations which introduced bias in favor of TMT. Among the single-center studies, the risk of selection bias was rated as "moderate" for the investigation of *Kim et al.* ($T_{0\,(TMT)}$: end of TMT, $T_{0\,(RC)}$: date of RC) [22] and "Ø information" for the studies of *Ikeda et al.* and *Nagao et al.* (definition of survival time missing) [20,21]. Furthermore, the investigation of *Smith et al.* [29] was rated as "moderate RoB" with regard to the domain "missing data" because one third of all patients had a missing comorbidity variable.
